# Supplementary material for: DMRforPairs: identifying Differentially Methylated Regions between unique samples using array based methylation profiles
Source: BMC Bioinformatics. 2014 May 15;15:141. doi: 10.1186/1471-2105-15-141 (PMC4046028; doi:10.1186/1471-2105-15-141)
Supplement: Additional file 2 — DMRforPairs output for the comparison of A431-MCF7 and NA17018-NA17105. Please start from the HTML files in each folder. Available via the BMC Bioinformatics website. [file 1471-2105-15-141-S2.zip › 1394847754114233_MOESM2_ESM/A431_MCF7/figures/19944.pdf]

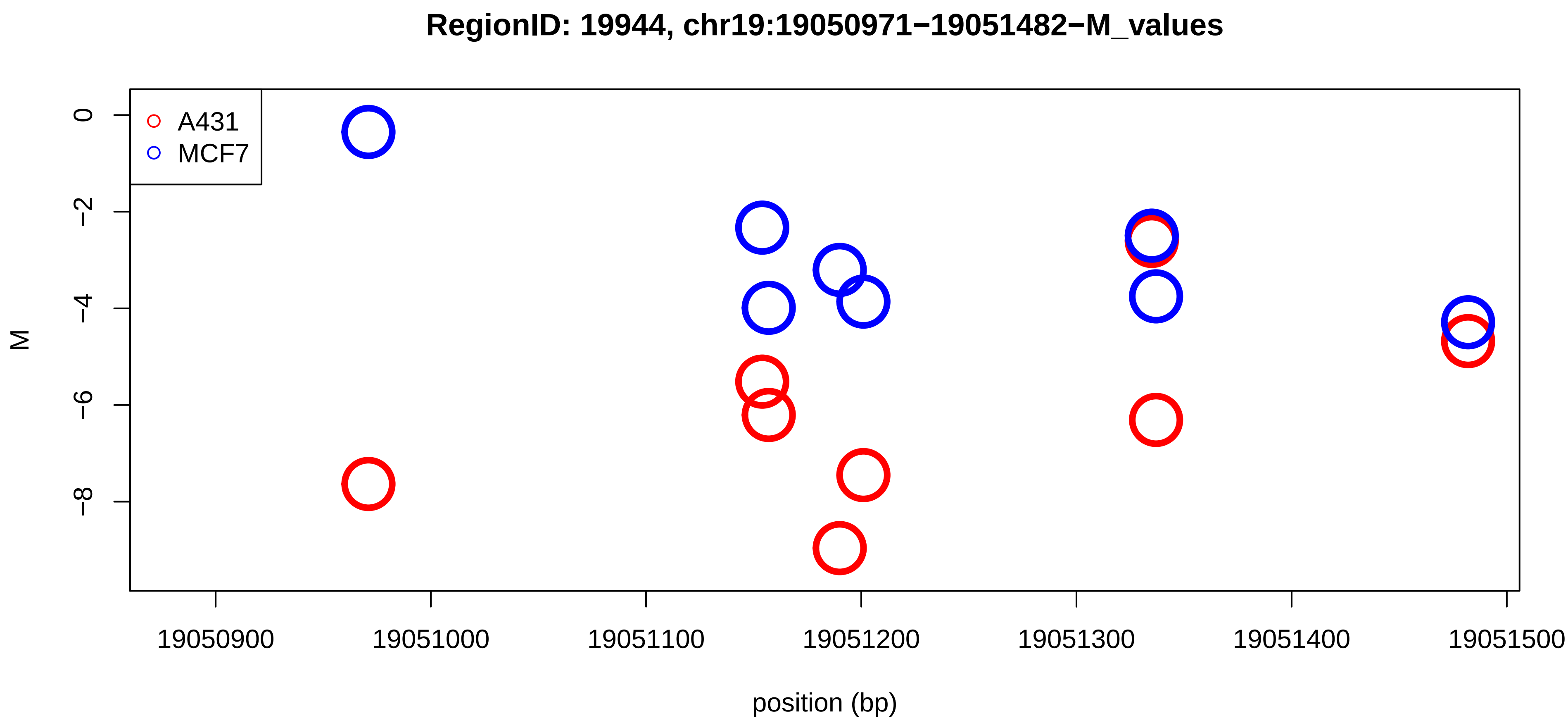
RegionID: 19944, chr19:19050971−19051482−M_values
A431
MCF7
M
0
−2
−4
−6
−8
19050900
19051000
19051100
19051200
19051300
19051400
19051500
position (bp)

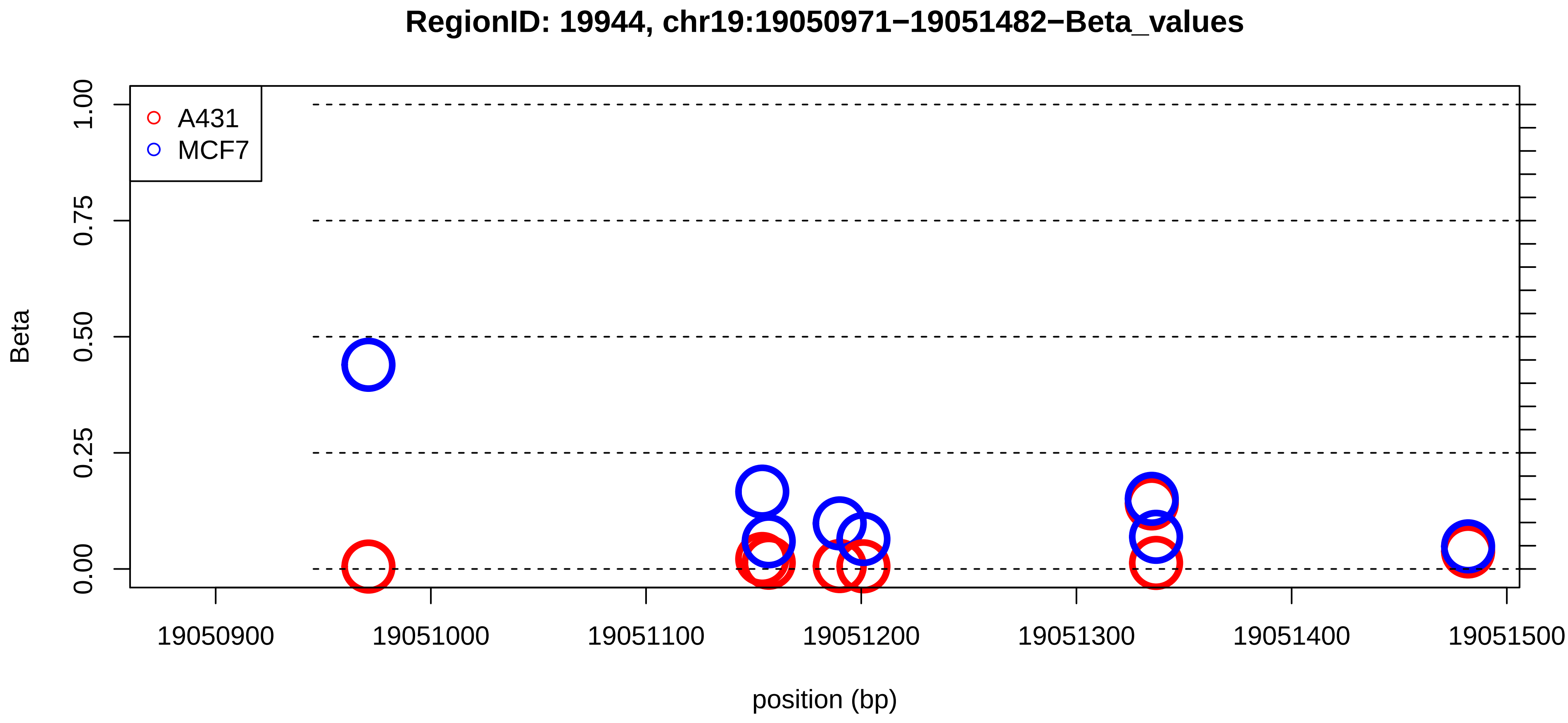
RegionID: 19944, chr19:19050971−19051482−Beta_values
A431
MCF7
Beta
1.00
0.75
0.50
0.25
0.00
19050900
19051000
19051100
19051200
19051300
19051400
19051500
position (bp)

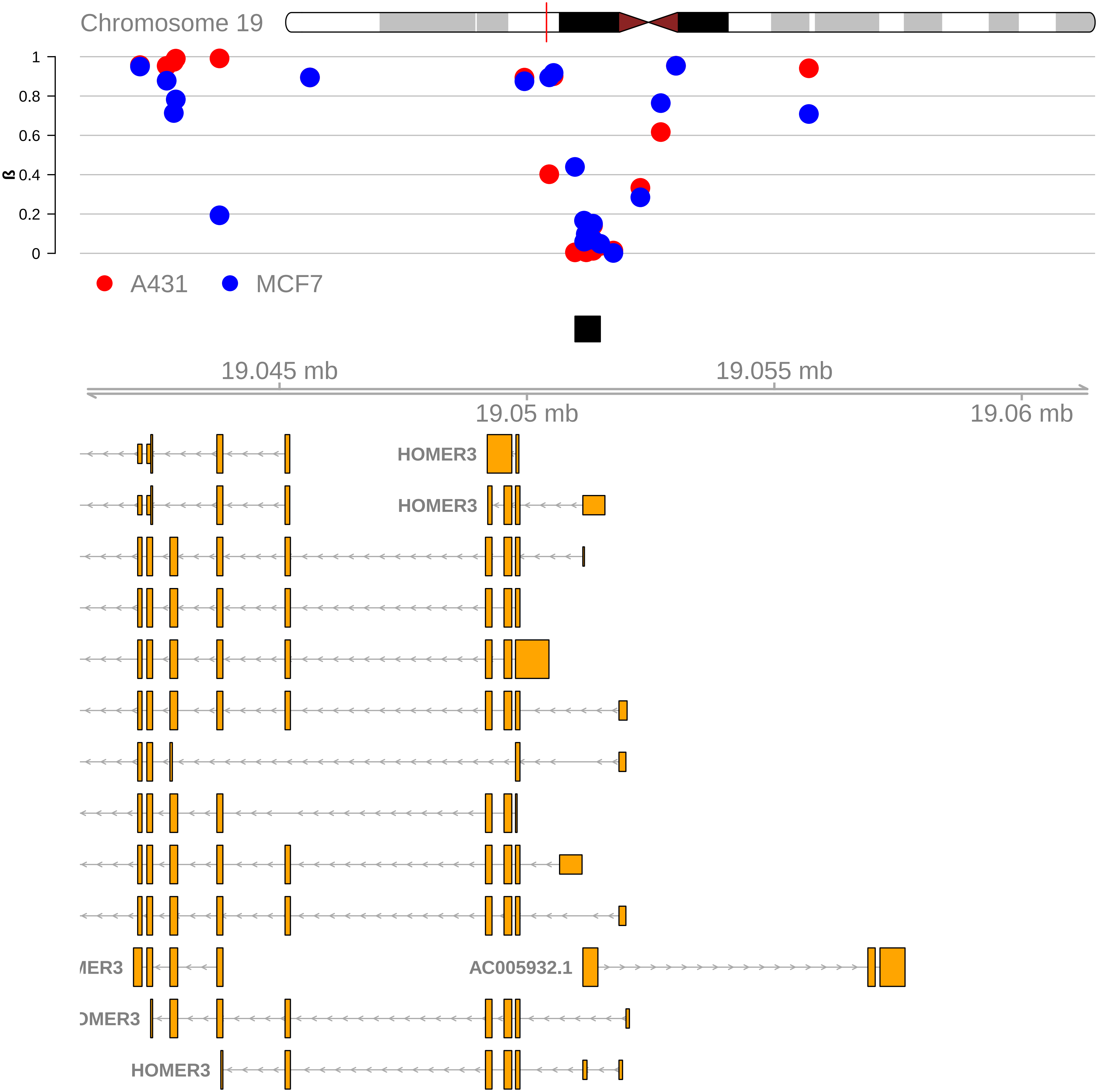

Chromosome 19
1
0.8
0.6
ß 0.4
0.2
0
A431
MCF7
19.045 mb
19.055 mb
19.05 mb
19.06 mb
HOMER3
HOMER3
MER3
AC005932.1
OMER3
HOMER3
